# Suppressive effect of α-mangostin for cancer stem cells in colorectal cancer via the Notch pathway

**DOI:** 10.1186/s12885-022-09414-6

**Published:** 2022-03-29

**Authors:** Min Kyoung Jo, Chang Mo Moon, Eun Ju Kim, Ji-Hee Kwon, Xiang Fei, Seong-Eun Kim, Sung-Ae Jung, Minsuk Kim, Yeung-Chul Mun, Young-Ho Ahn, Seung-Yong Seo, Tae Il Kim

**Affiliations:** 1 Department of Internal Medicine & Inflammation-Cancer Microenvironment Research Center, College of Medicine, Ewha Womans University, 1071 Anyangcheon-ro, Yangcheon-gu, Seoul, 07985 South Korea; 2 Inflammation-Cancer Microenvironment Research Center, College of Medicine, Ewha Womans University, Seoul, 07804 Republic of Korea; 3 Department of Molecular Medicine, College of Medicine, Ewha Womans University, Seoul, 07804 Republic of Korea; 4 Department of Internal Medicine, Yonsei University College of Medicine, 03722 Seoul, Republic of Korea; 5 College of Pharmacy, Gachon University, 191 Hambakmoe-ro, Yeonsu-gu, Incheon, 21936 Republic of Korea; 6 Department of Pharmacology, College of Medicine, Ewha Womans University, Seoul, 07804 Republic of Korea

**Keywords:** Cancer stem cell, Colorectal cancer, Notch signal, Phytochemical agent, α-Mangostin

## Abstract

**Background:**

Since colon cancer stem cells (CSCs) play an important role in chemoresistance and in tumor recurrence and metastasis, targeting of CSCs has emerged as a sophisticated strategy for cancer therapy. α-mangostin (αM) has been confirmed to have antiproliferative and apoptotic effects on cancer cells. This study aimed to evaluate the selective inhibition of αM on CSCs in colorectal cancer (CRC) and the suppressive effect on 5-fluorouracil (5-FU)-induced CSCs.

**Methods:**

The cell viability assay was performed to determine the optimal concentration of αM. A sphere forming assay and flow cytometry with CSC markers were carried out to evaluate the αM-mediated inhibition of CSCs. Western blot analysis and quantitative real-time PCR were performed to investigate the effects of αM on the Notch signaling pathway and colon CSCs. The in vivo anticancer efficacy of αM in combination with 5-FU was investigated using a xenograft mouse model.

**Results:**

αM inhibited the cell viability and reduced the number of spheres in HT29 and SW620 cells. αM treatment decreased CSCs and suppressed the 5-FU-induced an increase in CSCs on flow cytometry. αM markedly suppressed Notch1, NICD1, and Hes1 in the Notch signaling pathway in a time- and dose-dependent manner. Moreover, αM attenuated CSC markers CD44 and CD133, in a manner similar to that upon DAPT treatment, in HT29 cells. In xenograft mice, the tumor and CSC makers were suppressed in the αM group and in the αM group with 5-FU treatment.

**Conclusion:**

This study shows that low-dose αM inhibits CSCs in CRC and suppresses 5-FU–induced augmentation of CSCs via the Notch signaling pathway.

**Supplementary Information:**

The online version contains supplementary material available at 10.1186/s12885-022-09414-6.

## Background

CRC is the second-most frequent cause of cancer-related deaths in United States and many other high-income countries [1, 2]. While the best way to treat CRC is the complete surgical resection of the primary lesion, less than 25% of all patients are operable, and high percentage of patients may experience recurrence [3–6]. Patients with inoperable CRCs are usually treated with palliative chemotherapy, and a large number of patients have also required postsurgical chemotherapy for preventing tumor recurrence [7].

CSCs are small subset of the cancer cells with characteristics including proliferation, self-renewal, and asymmetric differentiation [8–10]. Conventional chemotherapeutic agents and radiotherapy may show therapeutic effects on rapidly growing tumors but cannot inhibit CSCs [11]. Previous studies reported that conventional chemotherapy can lead to an increase in colorectal CSCs [12, 13]. CSCs are closely associated with chemoresistance, cancer metastasis, and recurrence after primary therapy [8, 14–16]. Therefore, targeting of CSCs has emerged as an important aspect of effective cancer treatment.

Recently, certain components from fruit and vegetables were identified to have a chemopreventive effect on cancers and anticancer properties [17]. Among them, mangostin (*Garcinia mangostana*), a tropical evergreen tree commonly found in Southeast Asia [18–21], has been used in the traditional treatment of skin infections and in wound-healing for a long time [22]. Among the various secondary metabolites of mangostin, xanthones and polyphenolic substances show a variety of physiological activities including anti-inflammatory, antibacterial, and anticancer effects [23]. α-mangostin (αM) is one of the main bioactive and most abundant xanthones extracted from mangostin [23]. To date, αM has been widely investigated as a chemotherapeutic and chemopreventive bioactive compound [24]. In addition, novel xanthone derivatives based on αM were synthesized and evaluated as anticancer agents [25]. Consequently, αM has been shown to be effective in various cancers, including CRC, pancreatic, prostate, oral squamous, and breast cancers [18, 20, 21, 26–29]. In this study, we aimed to evaluate whether αM can selectively inhibit CSCs in CRC and whether it can also suppress an increase in the number of CSCs in combination with conventional anticancer agents.

## Methods

### Material

**Fig. 1:**
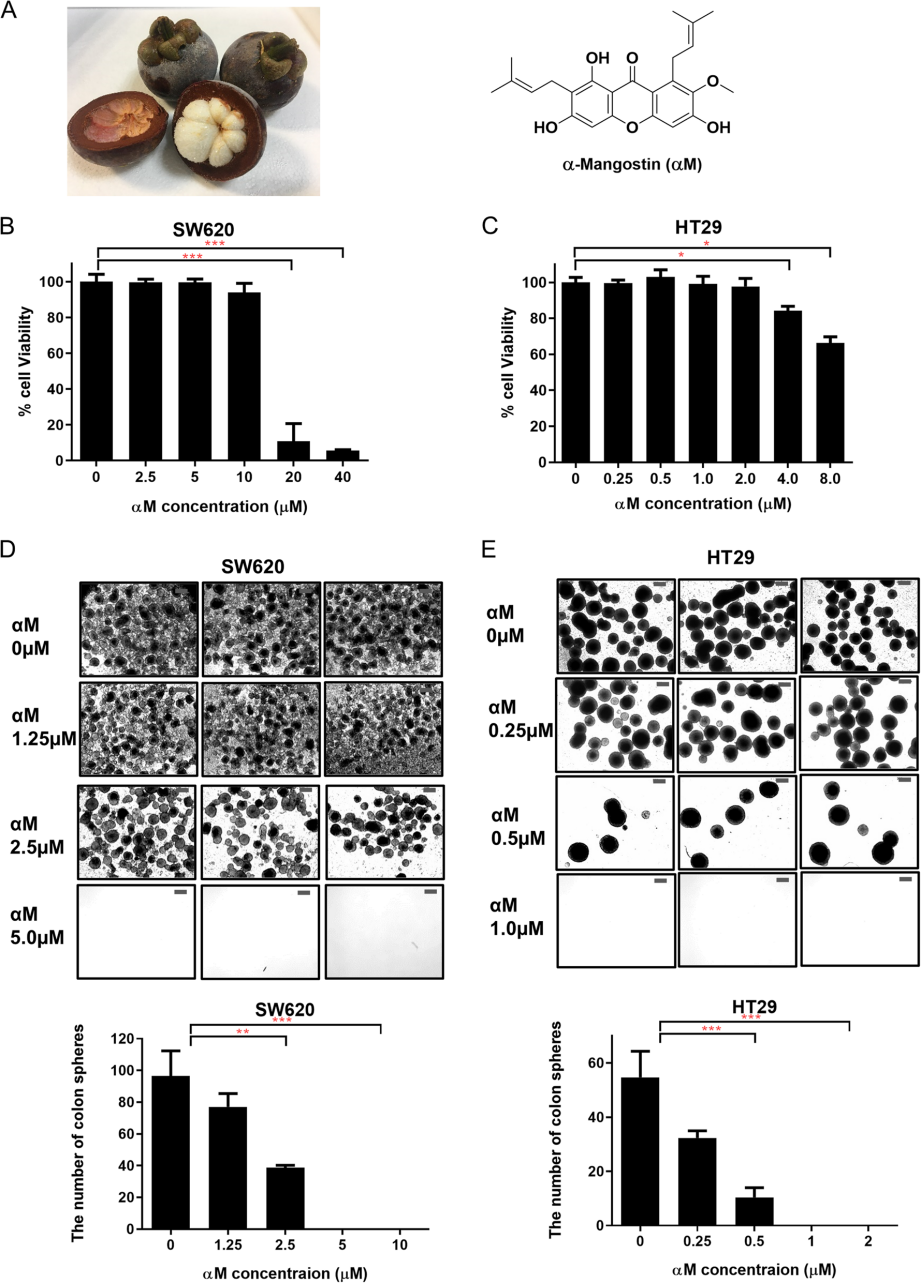
Cell viability assay and colosphere forming assay with αM–treated cancer stem cells. **A** Mangostin fruit and chemical structure of αM extracted from *Garcinia mangostana* Linn. **B**, **C** Effect of αM on the viability of SW620 and HT29 cells. SW620 and HT29 cells were treated with various concentrations of αM (0, 2.5, 5.0, 10, 20, and 40 μM in SW620 cells, *N* = 7; 0, 0.25, 0.5, 1.0, 2.0, 4.0, and 8.0 μM in HT29 cells, *N* = 4) for 72 h. **D**, **E** Colosphere-forming assay was performed with various concentrations of αM (0, 1.25, 2.5, 5, and 10 μM in SW620 cells; 0, 0.25, 0.5, 1, and 2 μM in HT29 cells) for 14 days. Based on a size-matched control for each cell line, the number of spheres in SW620 and HT29 cells were counted on day 14. *N* = 12 Data are expressed as mean ± SD values. ^*^P < 0.05, ^**^P < 0.01, ^***^P < 0.001

5-FU, dimethyl sulfoxide (DMSO), and N-[N-(3,5-difluorophenacetyl)-L-alanyl]-(S)-phenylglycine-t-butyl ester (DAPT) were purchased from Sigma-Aldrich (St. Louis, MO, USA). αM was provided by professor SY Seo (College of Pharmacy, Gachon University, Republic of Korea) (Fig. 1A). 5-FU and αM were dissolved in DMSO. The following antibodies were used for Western blotting and flow cytometry: anti-β-actin (1:1000, Gene Tex, Irvine, USA), anti-HES1 (1:1500, Cell Signaling, Danvers, MA, USA), anti-Notch1, anti-NICD 1 (1:100, Santa Cruz, TX, USA), anti-Hey1 (1:500, abcam, Cambridge, UK), fluorescein (FITC)-conjugated anti-CD44 (1:20, BD bioscience, Franklin Lakes, NJ), and phycoerythrin (PE)-conjugated anti-CD133 (1:50, Miltenyi Biotec, Bergisch Gladbach, Germany).

### Cell culture

Human colon cancer cell lines SW620 and HT29 were purchased from Korea Cell Line Bank (Seoul, Republic of Korea). Cells were cultured in Dulbecco’s modified Eagle medium (DMEM, Hyclone, Logan, UT, USA) supplemented with 10% fetal bovine serum (FBS, MP Biomedicals, France) and 1% antibiotic antimycotic solution (10,000 units/ml penicillin and 10 mg/ml streptomycin, Welgene, Daegu, Republic of Korea) in plastic tissue culture flasks under 37 °C, 5% CO_2_, and 95% humidity.

### Cell viability assay

Cell viability was measured by using Cell Counting Kit-8 (CCK-8, Enzo Life Sciences, Farmingdale, NY, USA). Cells were seeded in a 96-well plate (1 × 10^4^ cells/well, 200 μl/well, SPL, Republic of Korea) in an increasing gradient. SW620 cells were treated with 0, 2.5, 5, 10, 20, and 40 μM αM for 72 h, and HT29 cells were treated with 0, 0.25, 0.5, 1.0, 2.0, 4.0, and 8.0 μM αM. In each well, the medium was removed, and 90 μl plus 10 μl CCK-8 solution was added. Thereafter, the plate was incubated for 1 h at 37 °C. Absorbance was measured at 450 nm on a 96-well microplate reader (Spectra Max M5, MD, USA).

### Colosphere forming assay

SW620 and HT29 cells (1000 cells/well) were seeded in 24-well ultralow adherence plates (Corning, NY, USA) in 1 ml of CSC media, DMEM/F12 supplemented with B27 (Gibco, Invitrogen, Carlsbad, CA, USA), 2 mM L-glutamine (Hyclone), 10 ng/μl bFGF (Prospec, East Brunswick, NJ, USA), 20 ng/μl EGF (Prospec), and 1% antibiotic antimycotic solution (10,000 units/ml penicillin and 10 mg/ml streptomycin, Welgene). Cells were cultured for 14 d, and CSC medium was changed every 72 h. SW620 cells were treated with 0, 1.25, 2.5 μM αM, and HT29 cells were treated with 0, 1.25, 2.5 μM αM during the sphere forming assay. The spheres were examined using a microscope at 14 d (Zeiss Axiophot, Carl Zeiss Microscopy LLC, Thornwood, NY, USA). Quantitative real-time PCR and Western blot analyses were conducted with these cells.

### 3D spheroid invasion assay

The 3D spheroid invasion assay was conducted with the aforementioned HT29 cells. HT29 cells were trypsinized, and 1 × 10^5^ cells were resuspended in 5 mL DMEM with 20% methocel solution (methylcellulose, Sigma-Aldrich) and 1% Matrigel (Corning). Hanging drops (25 μl) were suspended on petri dishes (SPL), and cells were harvested after 2 d. Harvested spheroid cells were embedded in collagen gels (rat tail collagen, BD bioscience), which were polymerized at 37 °C. These spheroids were incubated for 5 d, and invasion ratios were calculated using ImageJ software (version 1.51 J8; National Institutes of Health, Bethesda, MD, USA).

### Quantitative real-time PCR analysis

Total cellular RNA was extracted from HT29 and SW620 cells, by using Trizol reagent (Invitrogen) and the RNeasy Mini Kit (Invitrogen) in accordance with the manufacturer’s protocol. The total RNA concentration was measured using a Nanodrop spectrophotometer (Nabi UV/Vis Nano spectrophotometer, Microdigital, Gyeonggi, Republic of Korea) with an A_260/280_ cut-off of approximately 2.0. Purified RNA (2 μg) was reverse-transcribed (with the Reverse Transcription Kit, Applied Biosystems, Framingham, MA, USA). Quantitative real-time PCR was performed with power SYBR Green master mix (Applied Biosystems) on Quant studio 3. The cycling conditions were as follows: denaturation for 2 min at 50 °C, 10 min at 95 °C, followed by 40 cycles at 95 °C for 5 s and 60 °C for 60 s, followed by dissociation for 15 s at 95 °C and annealing and extension at 60 °C for 20 s. The relative mRNA levels were normalized to those of β-actin mRNA using the 2^-ΔΔCt^ method. Primers for quantitative real-time PCR are listed in Supplementary Table S1.

### Western blot assay

The Western blot assay was conducted to determine the expression levels of Notch1, NICD1, Hes1, Hey1, and β-actin, under 4 experimental conditions. Proteins were extracted from cells by using radioimmunoprecipitation assay (RIPA) lysis buffer (iNtRON Biotechnology, Gyeonggi, Republic of Korea). The concentration of the isolated proteins was determined using a bicinchoninic acid (BCA) protein assay (Thermo Scientific-Pierce, Waltham, MA, USA). Proteins (20 μg) were separated through 8, 10, and 12% SDS-PAGE (Hoefer, San Francisco, CA, USA) and transferred to polyvinylidene fluoride membranes (PVDF, Merck). The membranes were blocked using 3% bovine serum albumin (BSA, Sigma-Aldrich) for 30 min at room temperature (RT). Protein extracts were incubated with primary antibodies overnight at 4 °C and with secondary antibodies for 1 h at RT. Proteins were detected using the enhanced chemiluminescence (ECL) Western blotting Luminol reagent (Santa Cruz). Images were obtained using a Lumino image analyzer (LAS-4000 Mini, Fujifilm, Tokyo, Japan).

### Flow cytometry analysis

For flow cytometry, cells were washed with PBS and incubated with Accutase (Gibco) for 10 min. After adding flow cytometry buffer (2.5 g BSA [Sigma-Aldrich] and 0.372 g EDTA [Sigma-Aldrich] in 500 ml PBS [Biosesang, Seongnam, Republic of Korea]), cells were incubated with primary antibodies at 4 °C in the dark for 45 min. CD133 was conjugated with PE and CD44 was conjugated with FITC for labeling cells. Labeled cells were resuspended in flow cytometry buffer. All samples were analyzed using the Novo-Cyte flow cytometer (ACEA Biosciences, San Diego, CA, USA).

### Assessment of in vivo anticancer efficacy

Six-week-old male Balb/c athymic mice were purchased from Orient Bio (Seongnam, Republic of Korea) and acclimated for 1 week. All mouse experiments were conducted under approved guidelines of the Animal Care and Use Committee of Ewha Womans University (EUM17-0368). HT29 cells (1 × 10^6^ cells) were suspended in DMEM with Matrigel matrix (1:1 ratio). The mixed cells were injected subcutaneously into the right rear flank of each mouse. After 11 days of injection, mice were divided into 4 treatment-based groups (5 mice per group): control, 5-FU only, αM only, and 5-FU and αM. 5-FU (30 mg/kg body weight) or/and αM (5 mg/kg) were administered intraperitoneally thrice a week for 18 d. Tumor volume was calculated (volume = length × width × width/2), and body weight was measured thrice a week. All mice were euthanized through CO_2_ asphyxiation, and the weight and volume of the excised tumor were measured on day 29.

### Statistical analysis

Data are expressed as mean ± standard error of the mean (SEM) or mean ± standard deviation (SD) values. All analyses were performed using Graph Pad Prism 8.0 software (Graph Pad Software, La Jolla, CA, USA) and SPSS software (version 22.0, Chicago, IL, USA). A *P* value of < 0.05 was considered significant. Statistical significance was determined using the Mann–Whitney *U* test for nonparametric data and a two-tailed Student *t* test for parametric data.

## Results

### Cell viability assay of αM-treated colon cancer cells

Figure 1A was shown the chemical structure of αM. Cell viability assays were performed to determine the minimum dose of αM, which can inhibit CSCs without obvious cytotoxicity. The cell viability of SW620 was 100% upon treatment with 0, 2.5, and 5 μM αM, 94.01% with 10 μM αM, 11.27% with 20 μM αM, and 5.73% with 40 μM αM (*P* < 0.001) (Fig. 1B). In HT29 cells, the cell viability was almost 100% upon treatment with 0–2.0 μM αM, and 84.16% with 4 μM αM (*P* < 0.05), and 66.26% with 8 μM αM (*P* < 0.05) (Fig. 1C). The results suggest that the optimal concentration of αM was less than 10 μM in SW620 cells and less than 2 μM in HT29 cells for further in vitro assays. Other CRC cells with a lower CSC proportion were SW480, DLD-1, and HCT116 cells, compared to SW620 and HT29 cells (Supplementary Fig. S1E). We also performed cell viability assay with αM on HT29, HCT116, DLD-1, and SW480 cells. The results showed that the inhibitory effect of αM was not concentration-dependent in HCT116, DLD-1, and SW480 cells. In addition, cell viability was suppressed by a higher dose of αM in HCT116, DLD-1, and SW480 cells compared to HT29 cells (Fig. 1C, Supplementary Fig. S1, S1B, S1C, S1D).

### Inhibitory effect of low-dose αM on colosphere formation

The number of spheres from SW620 cells decreased after the treatment with αM in a dose-dependent manner (Fig. 1D). Compared to the control group, 1.25 μM (*P* < 0.01) and 2.5 μM (*P* < 0.001) αM significantly decreased sphere formation in SW620 cells. In HT29 cells, the number of spheres were significantly decreased upon treatment with 0.25 μM (*P* < 0.001) and 0.5 μM (*P* < 0.001) αM (Fig. 1E). Sphere formation was not observed for SW620 cells treated with 5 and 10 μM αM and HT29 cells treated with 1 and 2 μM αM. These results indicate that sphere formation was suppressed with low-dose αM in both SW620 and HT29 cells.

### Reduction of CSCs and 5-FU–induced CSCs upon treatment with low-dose αM

**Fig. 2:**
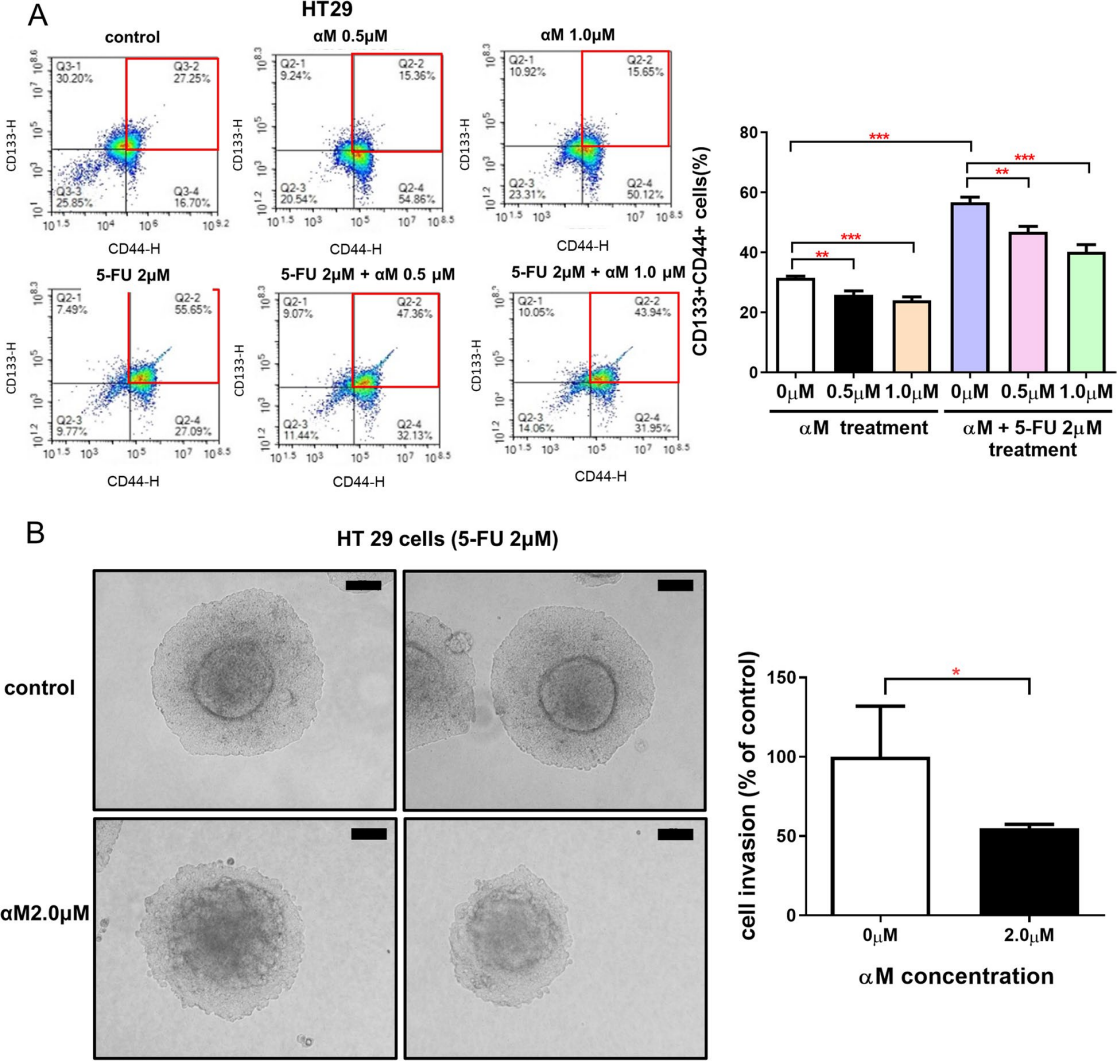
αM decreased CSCs and 5-FU–induced CSCs. **A** HT29 cells were treated with 0, 0.5, and 1.0 μM αM and with or without 2 μM 5-FU for 72 h. CD44-FITC and CD133-PE double-positive cells were analyzed using flow cytometry in HT29 cells. αM decreased the proportion of CD44 and CD133 cells relative to the control group, and αM with 5-FU treatment also reduced this proportion relative to 5-FU only. All data indicate dose-dependent effects. Data are expressed as mean ± SEM values. *N* = 10 (**B**) The 3D spheroid invasion assay was conducted to analyze the effect of αM on cancer cell invasion. αM significantly inhibited cancer cell invasion compared to the control group. Data are expressed as mean ± SD values. *N* = 5 ^*^P < 0.05, ^**^P < 0.01, ^***^P < 0.001

To evaluate the inhibitory effect of αM on CSCs and 5-FU–induced increase in CSCs, the expression levels of CD133 and CD44, which are well-known as CSC markers, were monitored after treating HT29 cells with αM with or without 5-FU for 72 h (Fig. 2A). The proportion of CD133^+^CD44^+^ cells significantly decreased upon treatment with 0.5 μM (control: 31.48% vs αM: 25.86%; *P* < 0.01) and 1.0 μM αM (control: 31.48% vs αM: 23.94%; *P* < 0.001). The proportion of CD133^+^CD44^+^ cells increased to 56.72% upon treatment with 2 μM 5-FU and decreased to 46.89% or 40.23% upon treatment with 0.5 μM or 1.0 μM of αM, respectively. The number of spheres from SW620 cells decreased after the treatment with 5-FU with or without αM (Supplementary Fig. S2A). These results suggest that αM selectively inhibits CSCs and the 5-FU–induced increase in CSCs. In addition, a 3D spheroid invasion assay was conducted to analyze the effect of αM on cancer cell invasion. As shown in Fig. 2B, αM significantly inhibited cancer cell invasion compared to the control (54.77% vs 100%, respectively; *P* < 0.05).

### Inhibition of CSCs via the NOTCH-HES1 pathway upon treatment with low-dose αM

**Fig. 3:**
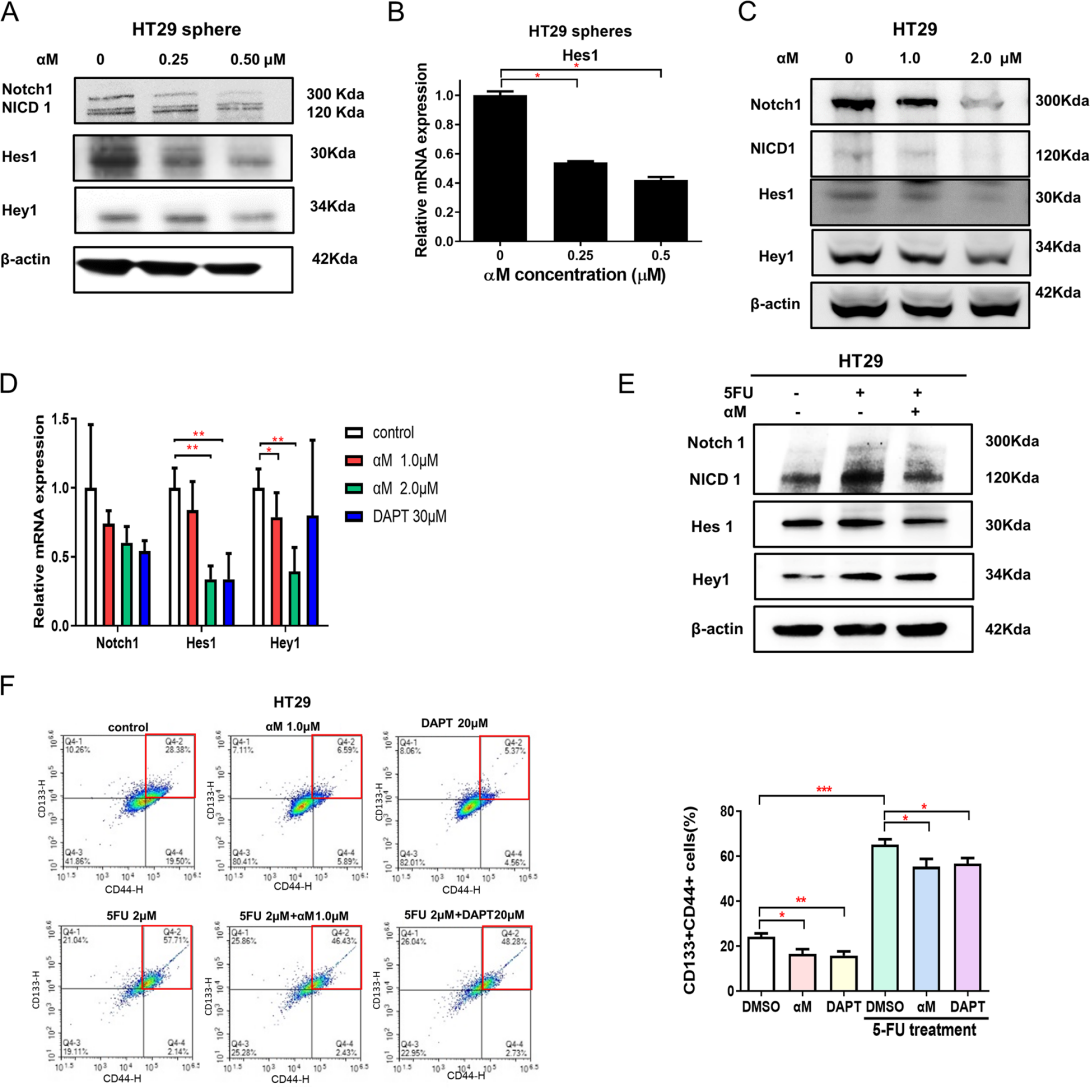
αM inhibited CSCs through the NOTCH-HES1 pathway. **A** Western blot showing protein levels of Notch1, NICD1, Hes1, Hey1, and β-actin in HT29 spheres treated with αM at different concentrations. Notch was downregulated upon treatment with 0, 0.25, and 0.5 μM αM in a concentration-dependent manner. **B** Quantitative real-time PCR showing the mRNA levels of Hes1 in HT29 spheres treated with 0, 0.25, and 0.5 μM αM for 14 d. Hes1 mRNA was downregulated following treatment with αM in sphere-forming assay. *N* = 3 (**C**) Western blot analysis for Notch1, NICD1, Hes1, Hey1, and β-actin with HT29 cells treated with αM at various concentrations. αM downregulated Notch1, NICD1, Hes1, and Hey1 in a concentration-dependent manner. **D** mRNA expression of Notch pathway factors: Notch1, Hes1, and Hey1 expression was quantified in HT29 cells through quantitative real-time PCR. αM downregulated Notch1, Hes1, and Hey1. *N* = 6 Data are expressed as mean ± SD values. **E** Western blot showing the protein levels of Notch1, NICD1, Hes1, Hey1, and β-actin in HT29 cells treated with or without 2 μM 5-FU and 1.0 μM αM. **F** Expression of CD44 and CD133 (CSC markers) was analyzed with or without 5-FU treatment through flow cytometry, using αM or DAPT. HT29 cells were treated with 1.0 μM αM and DAPT with or without 5-FU for 72 h for 11 times. The proportion of CD133^+^CD44^+^ cells was significantly decreased with both αM and DAPT. In addition, the 5-FU–induced increase in CD133^+^CD44^+^ cells was significantly attenuated by αM and DAPT treatment. *N* = 11 Data are expressed as mean ± SEM values; ^*^P < 0.05, ^**^P < 0.01, ^***^P < 0.001

Notch signaling, a highly conserved pathway, is reportedly involved in the self-renewal of CSCs and contributes to cancer metastasis (Gu et al., [30]; Pannuti et al., [31]). In colospheres of HT29 cells, treatment with 0.25 and 0.50 μM αM downregulated Notch1, Hes1, and Hey1 (Fig. 3A) and significantly attenuated Hes1 mRNA levels (Fig. 3B). Notch signaling proteins including Notch1, Hes1, and Hey1 were downregulated after αM treatment in HT29 and SW620 cells (Fig. 3C, Supplementary Fig. S2B). The mRNA levels of Hes1 (vehicle vs 2.0 μM αM, *P* = 0.002) and Hey1 (vehicle vs 1.0 μM αM, *P* = 0.026; vehicle vs 2.0 μM αM, *P* = 0.002) were downregulated following αM treatment, which was similar to the effect of treatment with the γ-secretase inhibitor DAPT (in Hes1, vehicle vs 30 μM DAPT, *P* = 0.002) (Fig. 3D). Notch signaling was upregulated with 5-FU treatment, and αM treatment attenuated the 5-FU-induced increase in Notch signaling in HT29 cells (Fig. 3E). This pattern was also observed in HT29 colosphere experiments (Supplementary Fig. S3B). As shown in Fig. 3F, the proportion CD133^+^CD44^+^ cells significantly decreased upon treatment with both αM (control: 25.26% vs 1.0 μM αM: 15.24%; *P* = 0.0083) and DAPT (control: 25.26% vs 20 μM DAPT: 15.36%; *P* = 0.0019). In addition, the 5-FU–induced increase in CD133^+^CD44^+^ cells were significantly attenuated upon treatment with αM (5-FU: 69.35% vs 5-FU + αM: 59.81%; *P* = 0.0473) and DAPT (5-FU: 69.35% vs 5-FU + DAPT: 57.36%; *P* = 0.0281). Furthermore, other signaling pathways related to CSCs, except for Notch signaling, were analyzed, which showed that they were not suppressed in a dose-dependent manner (Supplementary Fig. S4). These results suggest that Notch signaling is related to the CSC-suppressive effect of αM.

### CSC-inhibitory effect on αM in an in vivo xenograft mouse model

**Fig. 4:**
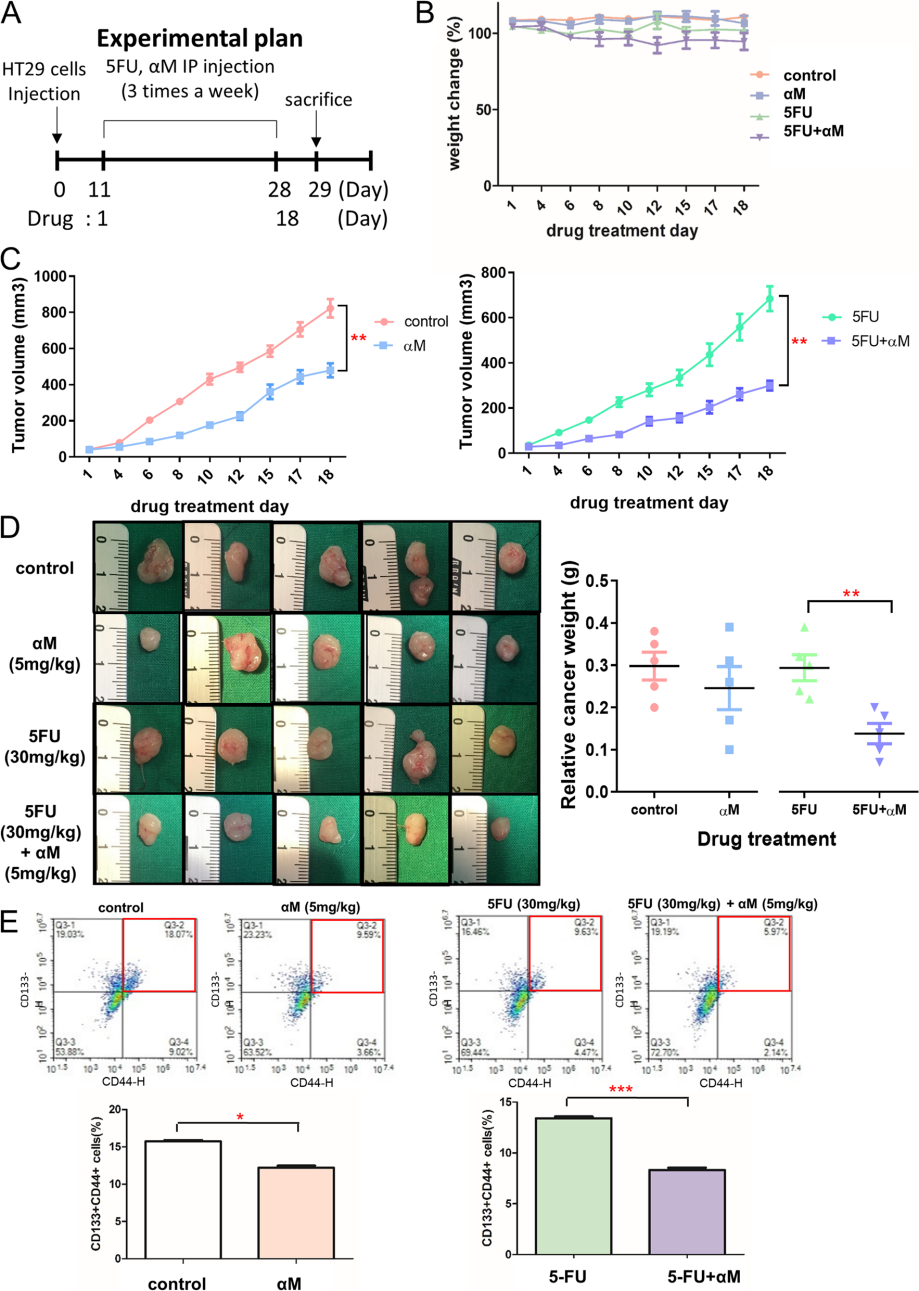
αM shows an inhibitory effect on CSCs in an in vivo xenograft mouse model. **A** Schematic representation of the experimental design. **B** Body weight of mice was not significantly different between the control group and the other groups during the experiment (Student’s *t* test). **C** Tumor volume in the HT29 xenograft mice treated with each agent. The tumor volume of the control group was larger than that in the αM group. Tumor volume of the 5-FU + αM group was significantly larger than that in the 5-FU only group. **D** Tumors from each group were weighed immediately after resection. The tumor weight in the 5-FU + αM group was significantly lower than that in the 5-FU group. **E** CD44/CD133 double-positive tumors in the αM group were significantly fewer than those in the αM group. These proportions were significantly lower in the 5-FU + αM group than in the 5-FU group. All Data is *N* = 5. Data are expressed as mean ± SEM values; ^*^P < 0.05, ^**^P < 0.01, ^***^P < 0.001

To evaluate the inhibitory effect of αM on CRC CSCs in vivo, its antitumor efficacy was assessed using a xenograft mouse model. 5-FU and/or αM were administered intraperitoneally to mice from days 11–28 (Fig. 4A). Among 4 groups including the control, 5-FU only, αM only, and 5-FU + αM, no significant differences were observed in body weight (Fig. 4B). Regarding tumor volume, tumors in the 5-FU + αM group were significantly smaller than those in the 5-FU only treatment group, and tumors in the αM group were significantly smaller than those in the control group (Fig. 4C). On day 29, the weight of the excised tumor of the 5-FU + αM group was significantly lower than that in the 5-FU only group (5-FU: 0.3 g vs 5-FU + αM: 0.14 g; *P* < 0.01), while no difference was observed between the control and αM only groups (Fig. 4D).

**Fig. 5:**
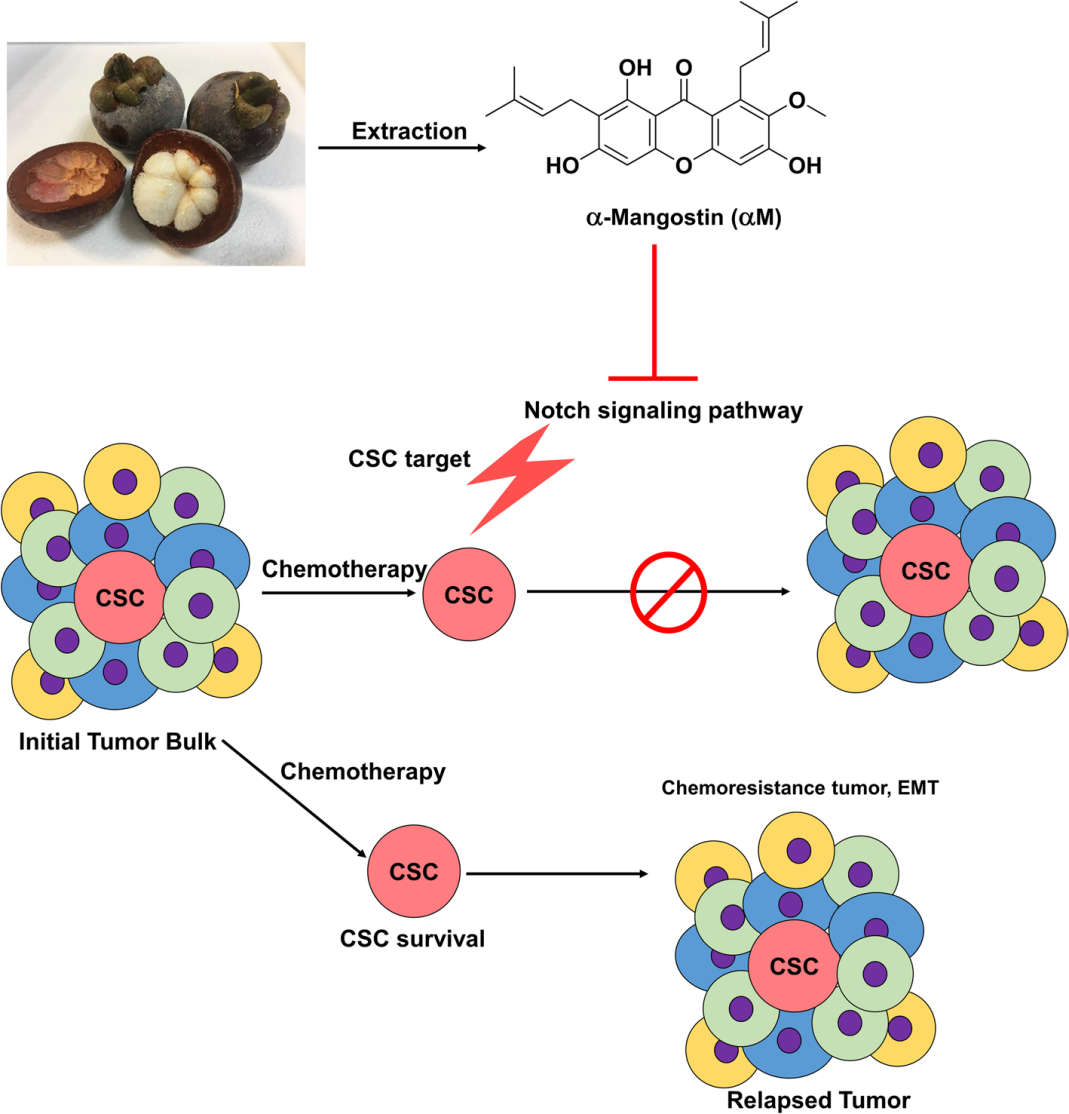
Schematic illustration of role of α-mangostin (αM) in regulation of CSCs in CRC cell lines. Low-dose of αM induced suppression of Notch signaling pathway in CRC, leading to a rise in targeting chemo-resistant CSCs

Regarding the CSC population in the excised tumors, treatment with αM only decreased the proportion of CD133^+^CD44^+^ cells compared to control (17.74% vs 11.72%, respectively; *P* < 0.01), and 5-FU + αM treatment attenuated these cells compared to the 5-FU only group (13.35 vs 8.39%, respectively; *P* < 0.001) (Fig. 4E). αM inhibits the notch signal pathway, leading the CSCs inhibition consequently (Fig. 5). Overall, our results show that αM not only inhibits CSCs but also exerts synergistic therapeutic effects in combination with 5-FU.

## Discussion

This study shows that αM has an inhibitory effect on CRC CSCs and attenuates a 5-FU–induced increase in CSCs. The effects of αM on apoptosis and cell cycle arrest through several signaling pathways in CRC have been widely studied [19, 32–35]. Several studies have reported that αM arrests the cell cycle by regulating cyclins and p27 in DLD-1 cells [33, 34]. Furthermore, αM induces apoptosis through the extrinsic and intrinsic pathways in COLO 205 cells [32, 35]. Moreover, αM induces apoptosis via the mitochondrial signaling pathway, which is regulated by MAPK, ERK, and Akt [19, 33]. In particular, apoptotic signals induced by the expression of proapoptotic proteins p21 and Bax owing to ERK activation, are relevant to the NF-κB pathway [19, 33].

Furthermore, αM is reported to have potential anticancer and antiproliferative effects on cervical and pancreatic CSCs [36, 37]. αM can inhibit CSC-like spheroids in human breast cancer cells, resulting in a significant reduction in the adherence and migration of cancer cells [38]. Combinatorial treatment with αM and cisplatin reportedly enhanced the therapeutic effects of cisplatin on cervical cancer and attenuated the chemoresistance of cancer cells to cisplatin by inducing apoptosis in CSC-like cervical cancer cells [36]. Combinatorial treatment with αM and doxorubicin reduces cell viability and decreases the retinaldehyde-dependent isoenzymes of aldehyde dehydrogenase (RALDH), a CSC marker, in spheroids of human breast cancer cell line MCF-7 [39]. Combinatorial treatment with αM and chemotherapeutic agents can help overcome chemoresistance through the suppression of CSCs.

The Notch signaling pathway, a highly conserved cellular signaling pathway, plays an important role in proliferation, stem cell maintenance, cell fate specification, differentiation, and homeostasis in multicellular organisms [40–42]. Some studies have reported that the Notch signaling is one of the most critical pathways in cancer metastasis [43]. Notch signaling can induce colon adenoma together with Wnt signaling and is necessary to eradicate drug-resistant CRC CSCs [44, 45]. Notch1 expression is dysregulated in the initiation step of CRC [46–48], positively predicts poor overall survival [49], and is upregulated in advanced tumors [50]. The inhibition of this pathway may enhance therapeutic efficacy in the curative treatment of cancer by eradicating CSCs [43, 51].

We hypothesized that αM selectively suppresses CSCs in CRC, and the combined use of αM and current anticancer agents including 5-FU exerts synergistic effects on CRC. The CSC-inhibitory effect was observed upon treatment with low-dose αM without concerns of cytotoxicity. Our results show the differences in αM concentrations and the sphere forming ability between the HT29 and SW620 cells. It should be noted that these differences originated from the different properties of each cell line. HT29 cells derived from primary cancer and SW620 cells were derived from lymph node metastasis [52]. Further, HT29 cells harbored V600E BRAF, P449T PIK3CA, R273H, and TP53 mutations, whereas SW620 cells harbored G12V KRAS, R273H, P309S, and TP53 mutations [52]. Even though both were CRC cell lines, the differences in experimental conditions were inevitable owing to intrinsic properties. In our study, the CSC proportion was around 10% for SW620 cells and 30% for HT29 cells. Accordingly, compared to HT29 cells, SW620 cells were inhibited with a higher concentration of αM in sphere-forming assay. This pattern was also observed in cell viability assay. Other CRC cells with a lower CSC proportion were SW480, DLD-1, and HCT116 cells compared to HT29 cells. In cell viability assay, the inhibitory effect of αM was not concentration-dependent in HCT116, DLD-1, and SW480 cells. In addition, cancer cell viability was suppressed by a higher dose of αM in HCT116, DLD-1, and SW480 cells compared to HT29 cells. These results support our hypothesis that αM may selectively inhibit CSCs in CRC.

In both HT29 spheres and the cell line, Notch, Hes1, and Hey1 were downregulated after αM treatment. In addition, the CSC proportion decreased upon treatment with both αM and DAPT. The RNA and protein levels of Notch1, NICD1, Hes1, and Hey1 were inhibited by αM in a concentration-dependent manner. Therefore, αM may inhibit the Notch signaling pathway at the transcriptional level. Of note, the mechanism underlying the CSC-inhibitory effects of αM in CRC are associated with Notch signaling. Moreover, the attenuation of the 5-FU–induced increase in CSCs by αM suggests that αM has the potential to suppress chemoresistance in CSCs. The 5-FU-induced increase in CSCs was suppressed with αM treatment. However, the CSC suppressive effect of αM was higher with αM treatment only than with 5-FU + αM treatment. Based on the results, when considering the clinical use of αM in CRC chemotherapy, the use of αM before conventional chemotherapeutic agents could have a greater therapeutic effect compared to the effect of simultaneous treatment.

## Conclusions

In conclusion, our results show that αM suppresses CSCs and inhibits the 5-FU–induced increase in CRC CSCs via Notch signaling. In particular, the fact that therapeutic efficacy is improved only with low-dose αM provides a strong advantage for clinical use. αM might be a promising adjunctive agent with conventional anticancer agents to improve treatment efficacy among patients with CRC.

## Supplementary Information

**Additional file 1.**

**Additional file 2.**

**Additional file 3.**

**Additional file 4.**

**Additional file 5.**

**Additional file 6.**

**Additional file 7.**

**Additional file 8.**

## Data Availability

All the data generated or analyzed during this study are included in this published article.

## Abbreviations

CRC: Colorectal cancer
CSC: Cancer stem cell
5-FU: Fluorouracil
DMEM: Dulbecco’s modified Eagle medium
FBS: Fetal Bovine Serum
DMSO: Dimethyl sulfoxide
Hes1: Hairy and enhancer of split
Hey1: Hes related with YRPW motif 1
FITC: Fluorescein
PE: Phycoerythrin
CCK-8: Cell Counting Kit-8
PVDF: Polyvinylidene fluoride membranes
RT: Room temperature
3D: Three dimension
ECL: Enhanced chemiluminescence
RIPA: Radioimmunoprecipitation assay
BSA: Bovine serum albumin
BCA: Bicinchoninic acid
