# Supplementary figures and images for: Suppressive effect of α-mangostin for cancer stem cells in colorectal cancer via the Notch pathway
Source: BMC Cancer. 2022 Mar 29;22:341. doi: 10.1186/s12885-022-09414-6 (PMC8962146; doi:10.1186/s12885-022-09414-6)

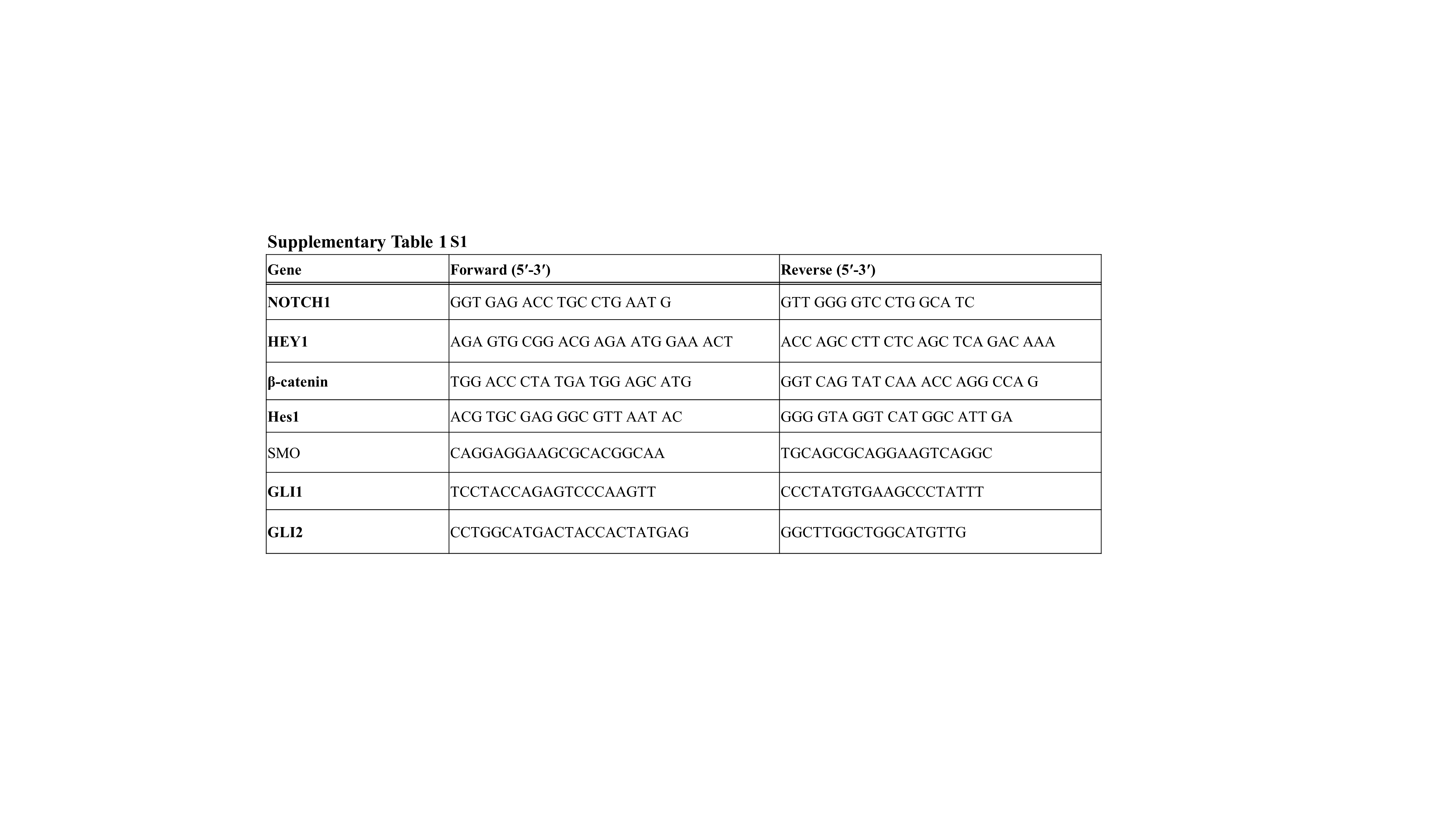

Supplement: Supplementary file 1 — Additional file 1. [file 12885_2022_9414_MOESM1_ESM.tif]

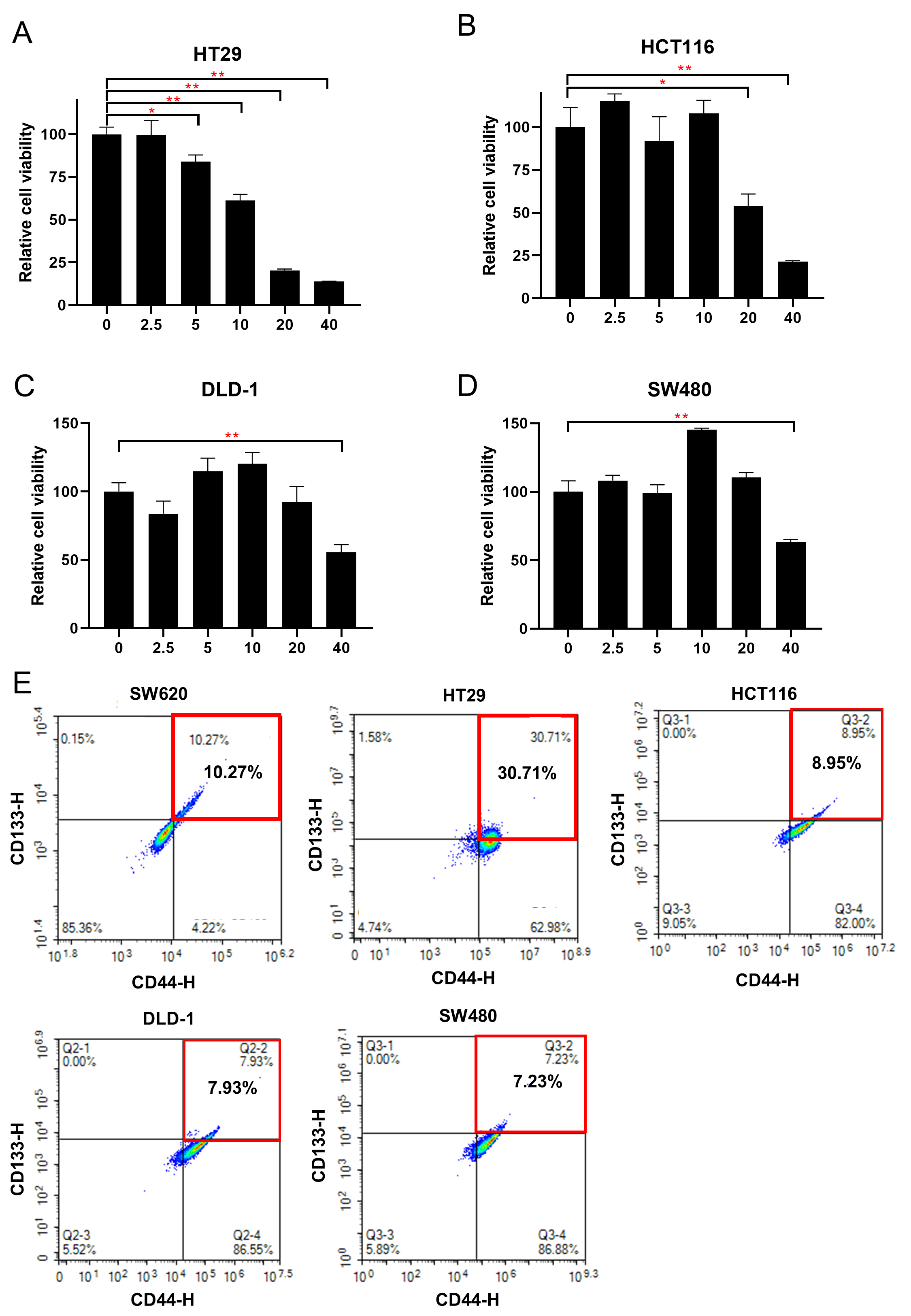

Supplement: Supplementary file 2 — Additional file 2. [file 12885_2022_9414_MOESM2_ESM.tif]

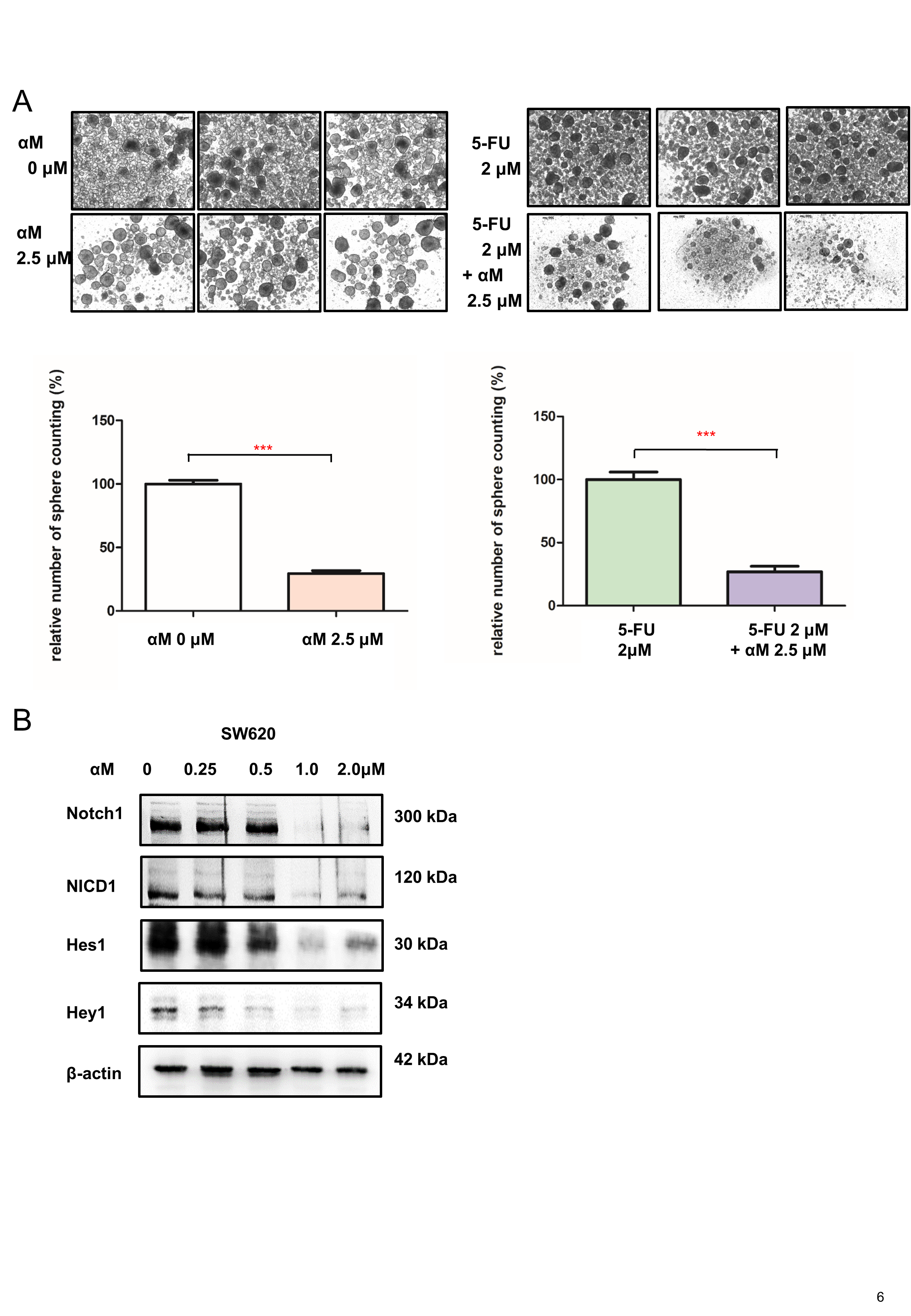

Supplement: Supplementary file 3 — Additional file 3. [file 12885_2022_9414_MOESM3_ESM.tif]

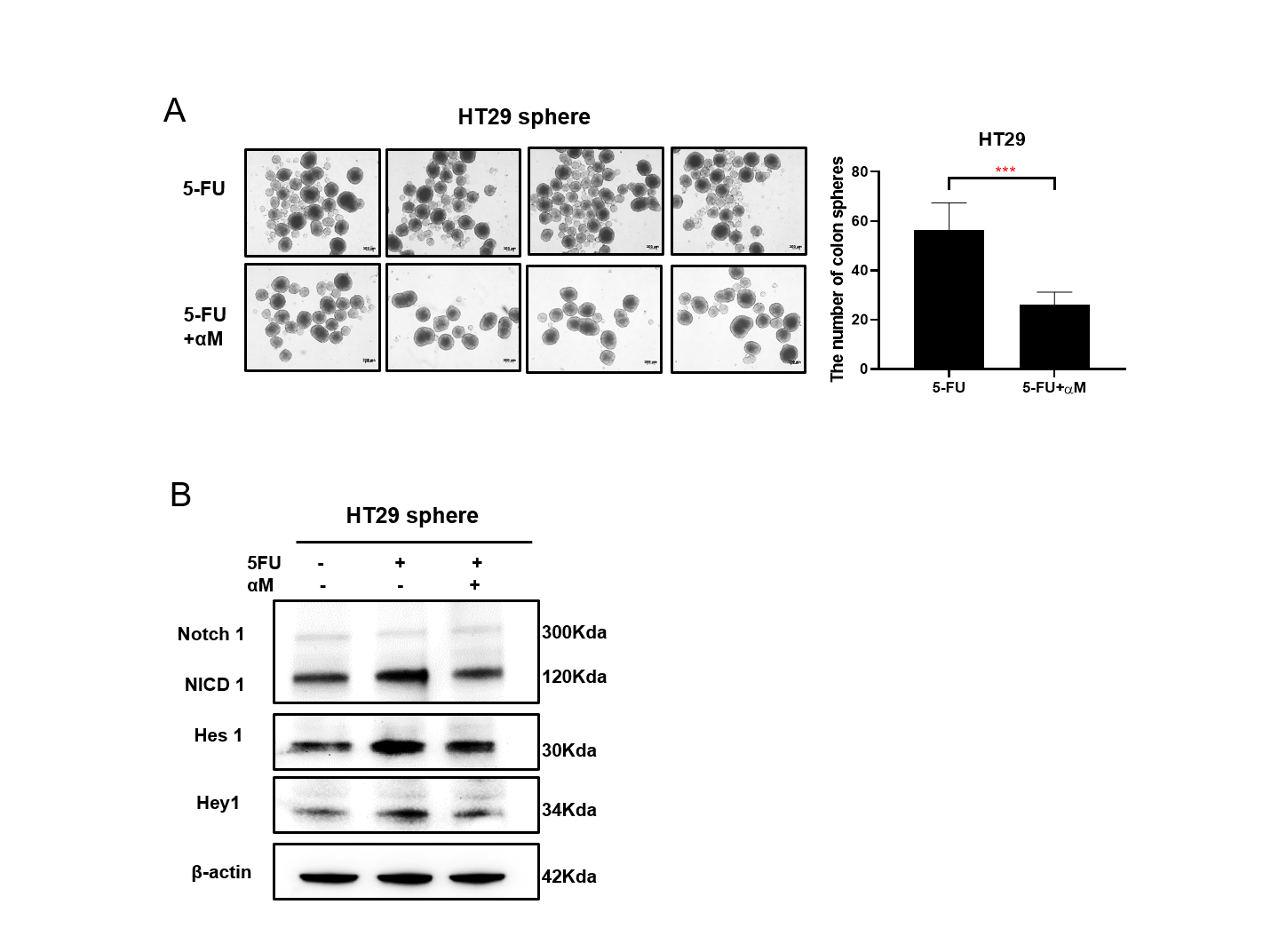

Supplement: Supplementary file 4 — Additional file 4. [file 12885_2022_9414_MOESM4_ESM.tif]

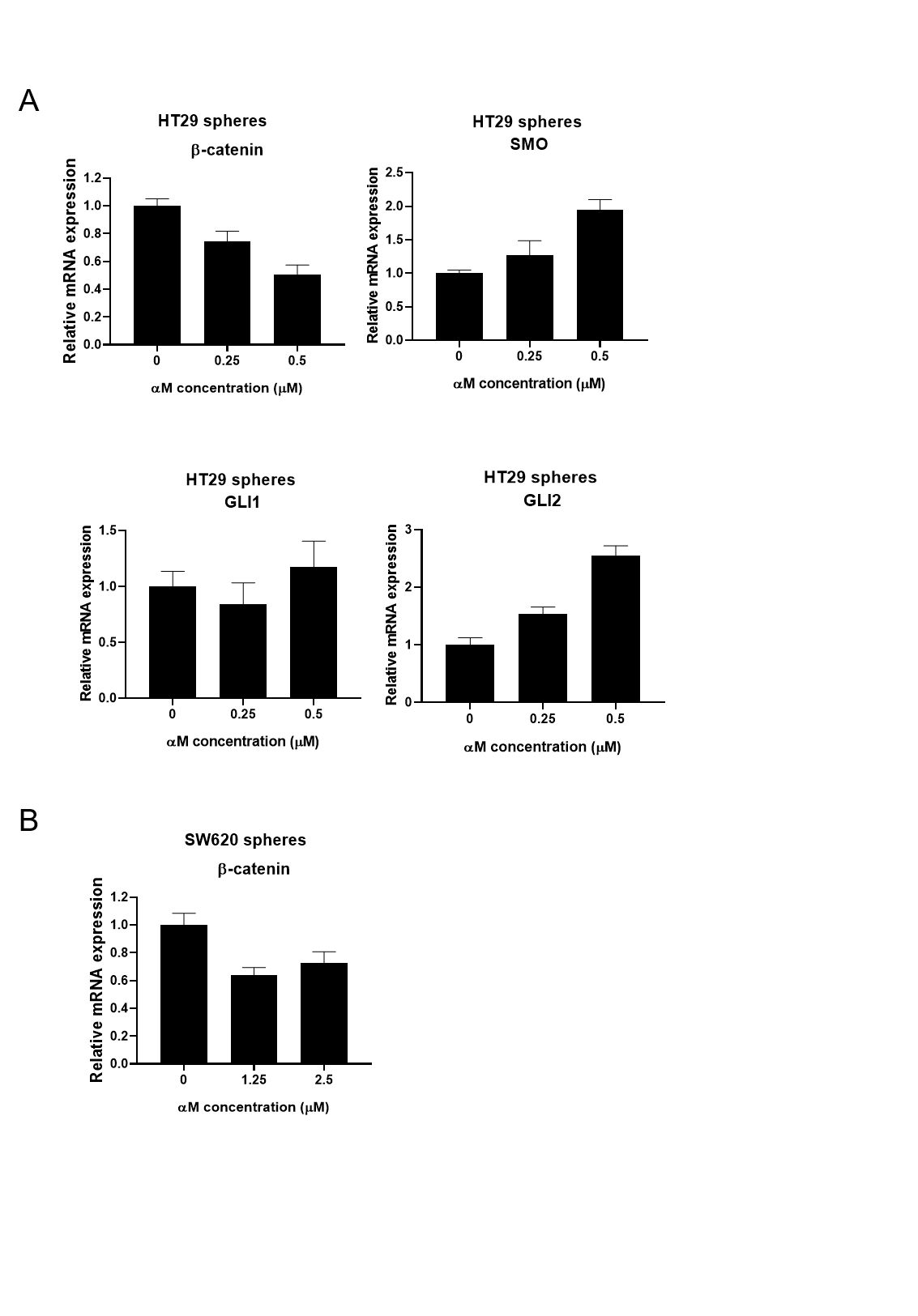

Supplement: Supplementary file 5 — Additional file 5. [file 12885_2022_9414_MOESM5_ESM.tif]

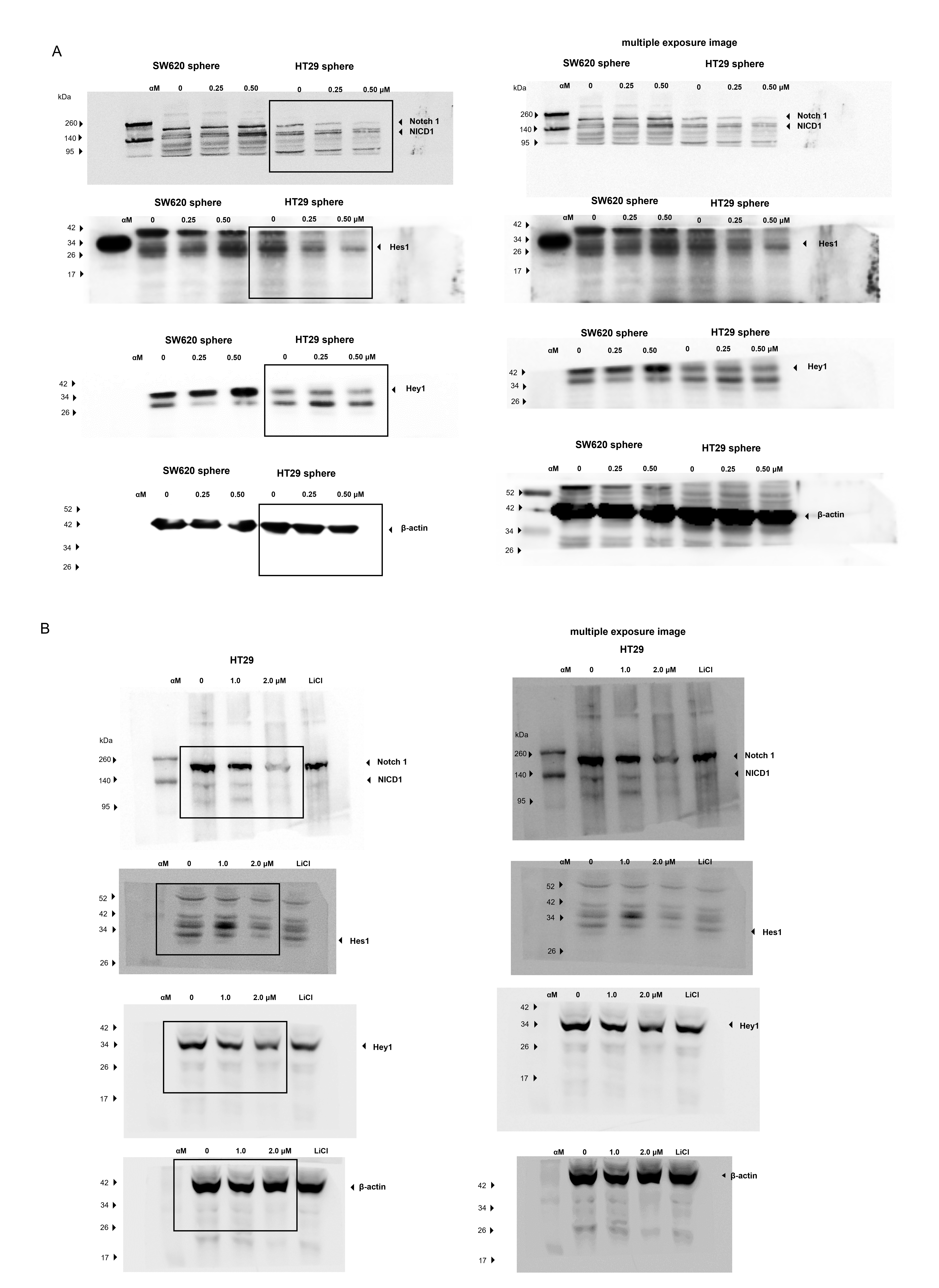

Supplement: Supplementary file 6 — Additional file 6. [file 12885_2022_9414_MOESM6_ESM.tif]

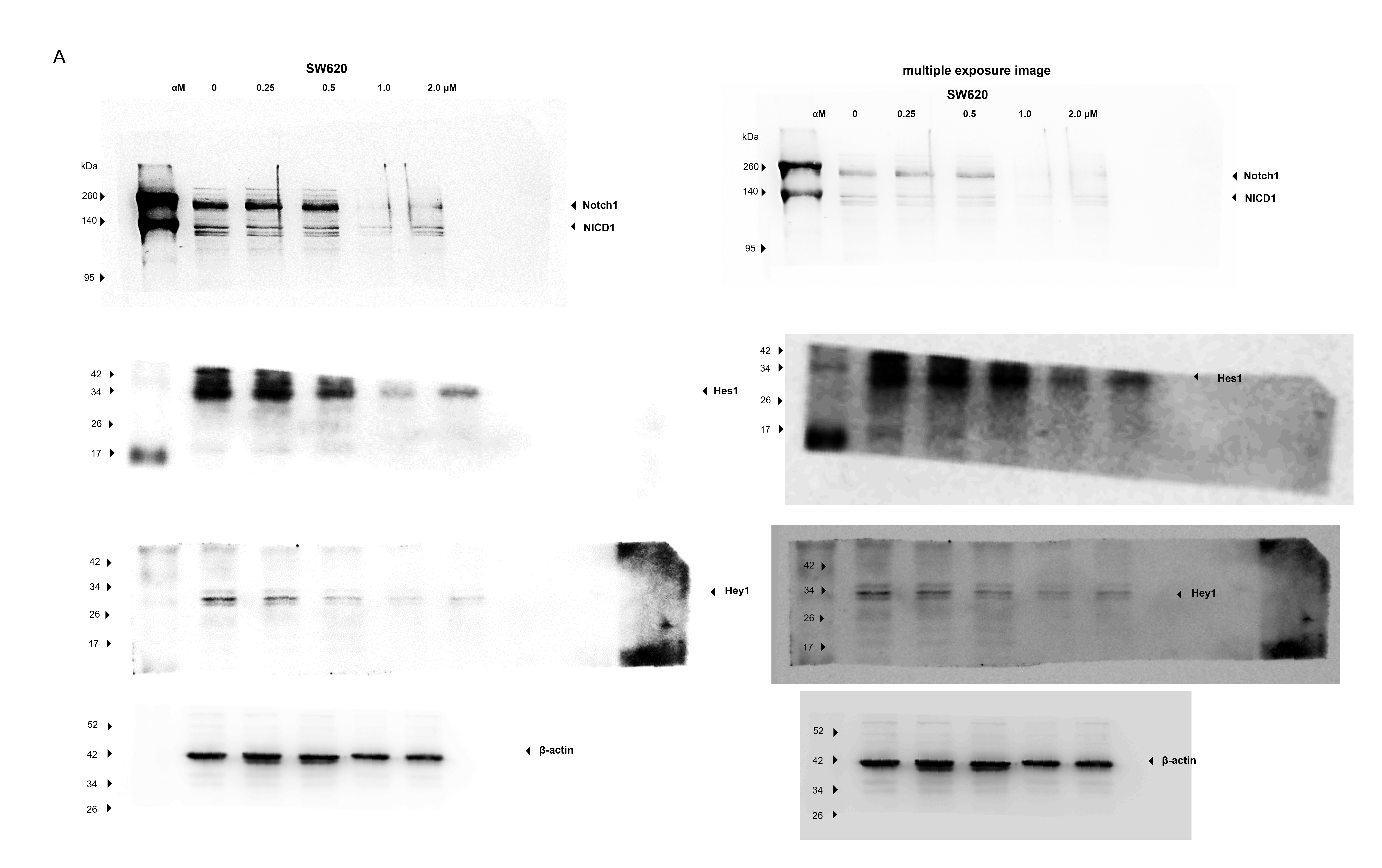

Supplement: Supplementary file 7 — Additional file 7. [file 12885_2022_9414_MOESM7_ESM.tif]

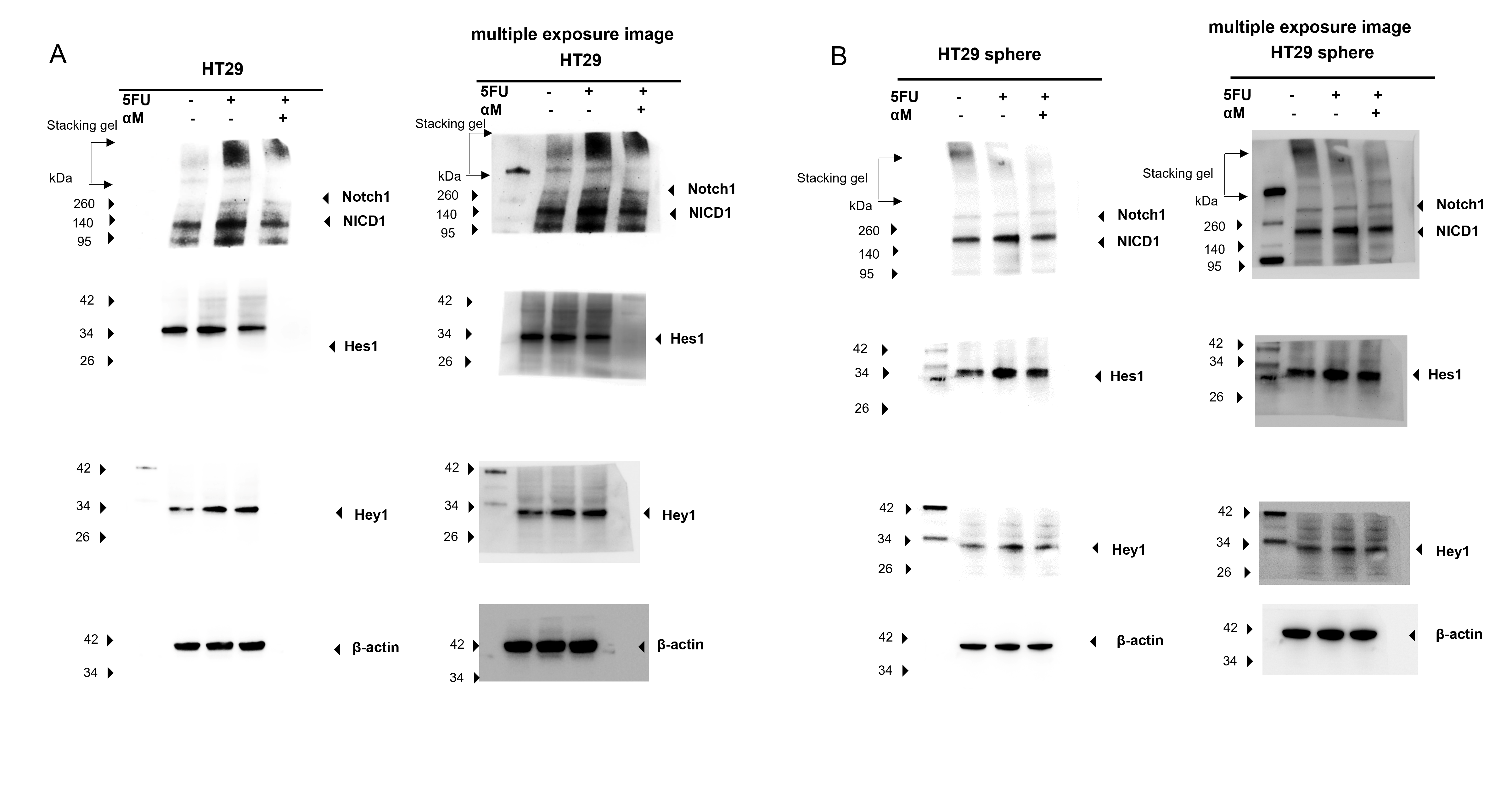

Supplement: Supplementary file 8 — Additional file 8. [file 12885_2022_9414_MOESM8_ESM.tif]
